# Supplementary material for: Demographic and socioeconomic characteristics associated with SARS-CoV-2 reinfection: An observational study
Source: PLOS Glob Public Health. 2026 Mar 10;6(3):e0006103. doi: 10.1371/journal.pgph.0006103 (PMC12974802; doi:10.1371/journal.pgph.0006103)
Supplement: S1 Table — (DOCX) [file pgph.0006103.s001.docx]

**S1 Table.** Risk factors for reinfection description, comparing the group of individuals that did not experience reinfections with the group that had reinfections.

|  | **No reinfections (n=7361)** | **1 or more reinfections (n=517)** |
| --- | --- | --- |
| **Age** |  |  |
| *Mean (SD)* | 37.8 (17.0) | 37.8 (14.9) |
| *Median (Min - Max)* | 36 (0 - 101) | 36 (1 - 87) |
| ***Sex*** |  |  |
| Females | 4176 (92.5%) | 339 (7.5%) |
| Males | 3185 (94.7%) | 178 (5.3%) |
| **Educational background** |  |  |
| *No education* | 253 (98.8%) | 3 (1.2%) |
| *Elementary school (complete or incomplete)* | 2588 (95.6%) | 120 (4.4%) |
| *High school (complete) or Vocational education* | 3517 (92.3%) | 293 (7.7%) |
| *Higher education/Postgraduate studies* | 1003 (90.9%) | 101 (9.2%) |
| **Monthly family income** |  |  |
| *No income* | 97 (87.4%) | 14 (12.6%) |
| *Up to 3 minimum wages (up to $552.66)* | 4913 (94.4%) | 294 (5.7%) |
| *3 to 6 minimum wages ($552.66 to $1,105.32)* | 2010 (92.0%) | 174 (8.0%) |
| *6 to 9 minimum wages ($1,105.32 to $1,657.98)* | 230 (88.5%) | 30 (11.5%) |
| *More than 9 minimum wages (Over $1,657.98)* | 111 (95.7%) | 5 (4.3%) |
| **Total house residents** |  |  |
| *0* | 510 (92.9%) | 39 (7.1%) |
| *1 or 2* | 3580 (93.3%) | 259 (6.8%) |
| *3 or 4* | 2737 (93.4%) | 192 (6.6%) |
| *5 or more* | 534 (95.2%) | 27 (4.8%) |
| **House size (number of rooms)** |  |  |
| *Up to 3* | 1039 (93.9%) | 68 (6.1%) |
| *4 or 5* | 4129 (93.0%) | 313 (7.1%) |
| *6 to 8* | 1921 (94.3%) | 116 (5.7%) |
| *9 or more* | 272 (93.2%) | 20 (6.9%) |
| **Protective measures: Face mask** |  |  |
| No | 99 (95.2%) | 5 (4.8%) |
| Yes | 7262 (93.4%) | 512 (6.6%) |
| **Protective measures: Social isolation** |  |  |
| No | 206 (94.9%) | 11 (5.1%) |
| Yes | 7155 (93.4%) | 506 (6.6%) |
| **Protective measures: Hand hygiene** |  |  |
| No | 3649 (92.6%) | 291 (7.4%) |
| Yes | 3712 (94.3%) | 226 (5.7%) |
| **Protective measures: None** |  |  |
| No | 7305 (93.4%) | 515 (6.6%) |
| Yes | 56 (96.6%) | 2 (3.5%) |
| **In the past two weeks, did you need to go to your workplace?** |  |  |
| No, I didn’t | 2716 (95.7%) | 122 (4.3%) |
| No, I worked from work | 128 (86.5%) | 20 (13.5%) |
| Yes, I worked on-site | 4517 (92.3%) | 375 (7.7%) |
| **Comorbidities*: Hypertension*** |  |  |
| No | 6365 (93.2%) | 463 (6.8%) |
| Yes | 996 (94.9%) | 54 (5.1%) |
| **Comorbidities*: Diabetes*** |  |  |
| No | 6916 (93.4%) | 486 (6.6%) |
| Yes | 445 (93.5%) | 31 (6.5%) |
| **Comorbidities*: Chronic heart disease*** |  |  |
| No | 7238 (93.4%) | 513 (6.6%) |
| Yes | 123 (96.9%) | 4 (3.2%) |
| **Comorbidities*: Chronic pulmonary disease (Asthma/POCD)*** |  |  |
| No | 7185 (93.5%) | 501 (6.5%) |
| Yes | 176 (91.7%) | 16 (8.3%) |
| **Comorbidities*: Chronic kidney disease stage 3, 4 and 5*** |  |  |
| No | 7319 (93.4%) | 517 (6.6%) |
| Yes | 42 (100.0%) | 0 (0.0%) |
