# Supplementary material for: Demographic and socioeconomic characteristics associated with SARS-CoV-2 reinfection: An observational study
Source: PLOS Glob Public Health. 2026 Mar 10;6(3):e0006103. doi: 10.1371/journal.pgph.0006103 (PMC12974802; doi:10.1371/journal.pgph.0006103)
Supplement: S2 Table — (DOCX) [file pgph.0006103.s002.docx]

**S2 Table:** Risk factors for reinfection (Poisson regression model with Robust Variance).

| **Comparisons** | **Relative Risk** | **Confidence Interval** | **p Value** | **Adjusted Relative Risk*** | **Confidence Interval** | **p Value**** |
| --- | --- | --- | --- | --- | --- | --- |
| *Age (each 1-year increase)* | 1.00 | 0.99-1.00 | 0.909 | 1.01 | 1.00-1.01 | 0.087 |
| *Gender (F vs M)* | 1.42 | 1.19-1.69 | <0.001 | 1.47 | 1.23-1.75 | <0.001 |
| *Educational background (Elementary school vs No education)* | 3.78 | 1.21-11.81 | 0.022 | 3.56 | 1.15-11.05 | 0.028 |
| *Educational background (High school or Vocational education vs No education)* | 6.56 | 2.12-20.32 | 0.001 | 5.67 | 1.83-17.56 | 0.003 |
| *Educational background (Higher education/Postgraduate studies vs No education)* | 7.81 | 2.50-24.42 | <0.001 | 5.85 | 1.84-18.55 | 0.003 |
| *Educational background (High school or Vocational education vs Elementary school)* | 1.74 | 1.41-2.13 | <0.001 | 1.59 | 1.29-1.96 | <0.001 |
| *Educational background (Higher education/Postgraduate studies vs Elementary school)* | 2.07 | 1.60-2.67 | <0.001 | 1.64 | 1.23-2.20 | <0.001 |
| *Educational background (Higher education/Postgraduate studies vs High school or Vocational education)* | 1.19 | 0.96-1.48 | 0.115 | 1.03 | 0.82-1.30 | 0.792 |
| *Family income (No income vs more than 9 minimum wages)* | 2.93 | 1.09-7.85 | 0.033 | 3.43 | 1.25-9.40 | 0.017 |
| *Family income (up to 3 minimum wages vs more than 9 minimum wages)* | 1.31 | 0.55-3.11 | 0.541 | 1.47 | 0.61-3.53 | 0.388 |
| *Family income (3 to 6 minimum wages vs more than 9 minimum wages)* | 1.85 | 0.78-4.41 | 0.166 | 1.89 | 0.79-4.49 | 0.151 |
| *Family income (6 to 9 minimum wages vs more than 9 minimum wages)* | 2.68 | 1.07-6.73 | 0.036 | 2.78 | 1.12-6.91 | 0.028 |
| *Family income (No income vs 6 to 9 minimum wages)* | 1.09 | 0.60-1.98 | 0.769 | 1.23 | 0.66-2.29 | 0.511 |
| *Family income (up to 3 minimum wages vs 6 to 9 minimum wages)* | 0.49 | 0.34-0.70 | <0.001 | 0.53 | 0.36-0.77 | 0.001 |
| *Family income (3 to 6 minimum wages vs 6 to 9 minimum wages)* | 0.69 | 0.48-0.99 | 0.047 | 0.68 | 0.47-0.98 | 0.040 |
| *Family income (No income vs 3 to 6 minimum wages)* | 1.58 | 0.95-2.64 | 0.078 | 1.82 | 1.07-3.09 | 0.028 |
| *Family income (up to 3 minimum wages vs 3 to 6 minimum wages)* | 0.71 | 0.59-0.85 | <0.001 | 0.78 | 0.64-0.95 | 0.014 |
| *Family income (No income vs up to 3 minimum wages)* | 2.23 | 1.35-3.69 | 0.002 | 2.33 | 1.39-3.90 | 0.001 |
| *House residents (0 vs 1 or 2)* | 1.05 | 0.76-1.46 | 0.755 | 1.12 | 0.80-1.55 | 0.510 |
| *House residents (0 vs 3 or 4)* | 1.08 | 0.78-1.51 | 0.635 | 1.12 | 0.79-1.58 | 0.529 |
| *House residents (0 vs 5 or more)* | 1.48 | 0.92-2.38 | 0.109 | 1.28 | 0.78-2.11 | 0.325 |
| *House residents (1 or 2 vs 3 or 4)* | 1.03 | 0.86-1.23 | 0.754 | 1.00 | 0.83-1.20 | 0.997 |
| *House residents (1 or 2 vs 5 or more)* | 1.40 | 0.95-2.06 | 0.087 | 1.15 | 0.77-1.70 | 0.491 |
| *House residents (3 or 4 vs 5 or more)* | 1.36 | 0.92-2.02 | 0.123 | 1.15 | 0.78-1.70 | 0.489 |
| *House size (up 3 vs 9 or more rooms)* | 0.90 | 0.55-1.45 | 0.658 | 1.05 | 0.62-1.76 | 0.859 |
| *House size (4 or 5 vs 9 or more rooms)* | 1.03 | 0.66-1.59 | 0.899 | 1.19 | 0.75-1.88 | 0.460 |
| *House size (6 to 8 vs 9 or more rooms)* | 0.83 | 0.53-1.32 | 0.430 | 0.89 | 0.56-1.42 | 0.631 |
| *House size (up to 3 vs 6 to 8 rooms)* | 1.08 | 0.81-1.44 | 0.609 | 1.18 | 0.86-1.60 | 0.310 |
| *House size (4 or 5 vs 6 to 8 rooms)* | 1.24 | 1.01-1.52 | 0.043 | 1.33 | 1.08-1.65 | 0.008 |
| *House size (up to 3 vs 4 or 5)* | 0.87 | 0.68-1.12 | 0.289 | 0.88 | 0.68-1.15 | 0.348 |
| *Protective measures: Face mask (No vs Yes)* | 0.73 | 0.31-1.72 | 0.473 | 0.83 | 0.32-2.13 | 0.701 |
| *Protective measures: Social isolation (No vs Yes)* | 0.77 | 0.43-1.37 | 0.373 | 0.72 | 0.38-1.35 | 0.301 |
| *Protective measures: Hand hygiene (No vs Yes)* | 1.29 | 1.09-1.52 | 0.003 | 1.35 | 1.14-1.60 | <0.001 |
| *Work in the last 2 weeks (Worked from home vs Didn’t work)* | 3.14 | 2.02-4.90 | <0.001 | 3.18 | 2.02-4.99 | <0.001 |
| *Work in the last 2 weeks (Worked on-site vs Didn’t work)* | 1.78 | 1.46-2.17 | <0.001 | 1.53 | 1.24-1.87 | <0.001 |
| *Work in the last 2 weeks (Worked on-site vs Worked from home)* | 0.57 | 0.37-0.86 | 0.008 | 0.48 | 0.31-0.74 | <0.001 |
| *HAS (Yes vs No)* | 0.76 | 0.58-1.00 | 0.048 | 0.80 | 0.60-1.07 | 0.136 |
| *Diabetes (Yes vs No)* | 0.99 | 0.70-1.41 | 0.964 | - |  | - |
| *Chronic heart disease (Yes vs No)* | 0.48 | 0.18-1.25 | 0.133 | - |  | - |
| *Chronic pulmonary disease (Yes vs No)* | 1.28 | 0.79-2.06 | 0.313 | - |  | - |
| *Chronic obstructive pulmonary disease exacerbated (Yes vs No)* | 2.78 | 0.79-9.76 | 0.111 | - |  | - |
| * Adjusted relative risks (RRs) were estimated using multivariable Poisson regression with robust variance, adjusted for demographic, socioeconomic, clinical, and behavioral covariates.  ** p-values were calculated using Student’s t-test for continuous variables (age) and Fisher’s exact test for categorical variables. | | | | | | |
