# Supplementary material for: Demographic and socioeconomic characteristics associated with SARS-CoV-2 reinfection: An observational study
Source: PLOS Glob Public Health. 2026 Mar 10;6(3):e0006103. doi: 10.1371/journal.pgph.0006103 (PMC12974802; doi:10.1371/journal.pgph.0006103)
Supplement: S3 Table — (DOCX) [file pgph.0006103.s003.docx]

**S3 Table:** Adherence to quarantine preventive measures comparison in the population with only one infection and the population with reinfections.

|  | **1st infection** | **2nd infection** | **3rd infection** |
| --- | --- | --- | --- |
| **Protective measures: Face mask *(n=8483)*** |  |  |  |
| No | 103 (1.3%) | 5 (0.9%) | 1 (5.0%) |
| Yes | 7823 (98.7%) | 532 (99.1%) | 19 (95.0%) |
| **Protective measures: Social isolation *(n=8483)*** |  |  |  |
| No | 221 (2.8%) | 12 (2.2%) | 0 (0.0%) |
| Yes | 7705 (97.2%) | 525 (97.8%) | 20 (100.0%) |
| **Protective measures: Hand hygiene *(n=8483)*** |  |  |  |
| No | 3862 (48.7%) | 304 (56.6%) | 15 (75.0%) |
| Yes | 4064 (51.3%) | 233 (43.4%) | 5 (25.0%) |
| **In the past two weeks, did you need to go to your workplace? *(n=8483)*** |  |  |  |
| No | 3024 (38.1%) | 150 (27.9%) | 5 (25.0%) |
| Yes | 4904 (61.9%) | 388 (72.1%) | 15 (75.0%) |
