# Supplementary material for: Demographic and socioeconomic characteristics associated with SARS-CoV-2 reinfection: An observational study
Source: PLOS Glob Public Health. 2026 Mar 10;6(3):e0006103. doi: 10.1371/journal.pgph.0006103 (PMC12974802; doi:10.1371/journal.pgph.0006103)
Supplement: S4 Table — (DOCX) [file pgph.0006103.s004.docx]

**S4 Table:** Preventive measures adherence (Adjusted prevalence ratio)

|  | **Adjusted prevalence ratio *** | **95% Confidence Interval** | **p Value** |
| --- | --- | --- | --- |
| **Protective measures: Face mask** |  |  |  |
| *Infection (2 vs 1)* | 1.003 | 0.994-1.012 | 0.487 |
| *Infection (3 vs 1)* | 0.962 | 0.870-1.064 | 0.448 |
| *Infection (3 vs 2)* | 0.959 | 0.867-1.061 | 0.415 |
| **Protective measures: Social isolation** |  |  |  |
| *Infection (2 vs 1)* | 1.007 | 0.994-1.021 | 0.310 |
| *Infection (3 vs 1)* | 1.032 | 1.024-1.040 | <0.001 |
| *Infection (3 vs 2)* | 1.025 | 1.011-1.040 | <0.001 |
| **Protective measures: Hand hygiene** |  |  |  |
| *Infection (2 vs 1)* | 0.829 | 0.752-0.914 | <0.001 |
| *Infection (3 vs 1)* | 0.466 | 0.227-0.956 | 0.037 |
| *Infection (3 vs 2)* | 0.562 | 0.272-1.162 | 0.120 |
| **In the past two weeks, did you need to go to your workplace?** |  |  |  |
| *Infection (2 vs 1)* | 1.094 | 1.039-1.152 | <0.001 |
| *Infection (3 vs 1)* | 1.186 | 0.940-1.496 | 0.150 |
| *Infection (3 vs 2)* | 1.084 | 0.856-1.372 | 0.502 |
| * Adjusted for age, gender, education background, family income, number of house residents, house size, and comorbidities (hypertension). | | | |
