# Supplementary material for: Demographic and socioeconomic characteristics associated with SARS-CoV-2 reinfection: An observational study
Source: PLOS Glob Public Health. 2026 Mar 10;6(3):e0006103. doi: 10.1371/journal.pgph.0006103 (PMC12974802; doi:10.1371/journal.pgph.0006103)
Supplement: S5 Table — (DOCX) [file pgph.0006103.s005.docx]

**S5 Table:** Relative risk for severe infections, with score ≥4 on the WHO Clinical Progression Scale (Poisson regression model with Robust Variance).

|  | **Adjusted relative risk *** | **95% Confidence Interval** | **p Value** |
| --- | --- | --- | --- |
| *Infection (1 vs 2)* | 2.118 | 1.342-3.343 | 0.001 |
| *Infection (1 vs 3)* | 1.042 | 0.145-7.497 | 0.967 |
| *Infection (2 vs 3)* | 0.492 | 0.065-3.752 | 0.494 |
| *Adjusted for age, gender, and comorbidities (hypertension and diabetes). | | | |
