# Supplementary material for: Demographic and socioeconomic characteristics associated with SARS-CoV-2 reinfection: An observational study
Source: PLOS Glob Public Health. 2026 Mar 10;6(3):e0006103. doi: 10.1371/journal.pgph.0006103 (PMC12974802; doi:10.1371/journal.pgph.0006103)
Supplement: Attachment — Submitted filename: Response to Reviewers.docx [file pgph.0006103.s007.docx]

September 16, 2025

Dr. Catherine Kyobutungi and Professor Madhukar Pai

Editor-in-Chief

*PLOS Global Public Health*

Re: PGPH-D-24-03019 - Clinical, epidemiological, and socioeconomic characteristics associated with SARS-CoV-2 reinfection: an observational study.

Dear Dr. Kyobutungi and Professor Pai,

We thank you and the referees for the review of our manuscript. We have made the necessary changes to a new version of the manuscript accordingly. The answers and changes are detailed below and are highlighted in the manuscript.

**Journal Requirements:**

**Requirement 1:** Please amend your detailed Financial Disclosure statement. It must be completed in full sentences and contain the exact wording you wish to be published. If you did not receive any funding for this study, please simply state: “The authors received no specific funding for this work.”

**Response:** Thank you for pointing that out. Since the authors did not receive any specific funding for this study, we have included the following statement in the manuscript: "The authors received no specific funding for this work”.

**Requirement 2:** Please note that your Data Availability Statement is currently missing the repository name. If your manuscript is accepted for publication, you will be asked to provide these details on a very short timeline. We therefore suggest that you provide this information now, though we will not hold up the peer review process if you are unable.

**Response:** We appreciate your observation regarding the Data Availability Statement. We have now included the name of the repository and the DOI for the dataset. The data underlying the findings of this study have been deposited in the public repository Zenodo and can be accessed at the following DOI: https://doi.org/10.5281/zenodo.14541548.

**Requirement 3:** If the reviewer comments include a recommendation to cite specific previously published works, please review and evaluate these publications to determine whether they are relevant and should be cited. There is no requirement to cite these works unless the editor has indicated otherwise.

**Response:** Thank you for the clarification. None of the reviewers recommended citing any specific previously published works.

**Comment 1:** Please change 'descriptive and observational cohort' to 'cohort' as cohorts are observational studies by design. Also, kindly highlight how reinfections were identified - was it solely based on medical records or was any active follow-up conducted? If later, what was the frequency & duration of follow-up, & how many participants could the researchers contact during the follow-up period (i.e., was there any loss to follow-up)? If reinfections were identified through patient medical records, is there a possibility of reporting bias (i.e., some reinfections were missed as participants did not report to the hospital/undergo testing)?

**Response:** We thank you for this important comment. Reinfections were identified through official database medical records. It is important to note that in Brazil all COVID-19 cases had to be reported in the national official databases (e-SUS and SIVEP-Gripe). Additionally, in Serrana, an enhanced surveillance program for COVID-19 cases was implemented, further improving case detection. Anyone presenting with one or more symptoms for at least two days could access a free RT-PCR nasal swab test, with results available the following working day. Positive SARS-CoV-2 samples were subsequently sequenced and patients were actively followed for 28 days or until hospital discharge or death. After this period, no further follow-up was conducted; therefore reinfections were identified only if individuals returned to health services and tested positive again. While most cases were likely captured given the accessibility of free testing and the mandatory reporting system, we acknowledge the possibility that some reinfections may have been missed if individuals did not seek testing. We have clarified this information and acknowledged this as a limitation of our study.

**Comment 2:** Please clarify how the epidemiological risk-factor data was collected. Was it through a questionnaire or was it based on patient medical records? If through a questionnaire, was the questionnaire self-reported or interviewer administered? How was the questionnaire developed & validated? Also, when was the risk-factor data obtained? Was it at the time of initial COVID-19 diagnosis or was it at the time of diagnosis of reinfection? If the risk-factor data was obtained at the time of initial infection, is there any chance of misclassification bias (esp. for the preventive measures as the practices such as mask wearing or handwashing may change over time)?

**Response:** We thank you for this comment. Epidemiological and risk-factor data were obtained through a structured form routinely completed by the epidemiological surveillance nursing staff at the time of SARS-CoV-2 diagnostic testing. This form is part of the official medical record, developed by the local epidemiological surveillance department and reviewed by public health and infectious disease specialists. Data were collected at each testing event; therefore, individuals who experienced reinfection underwent multiple tests and responded to the same set of questions on each occasion, allowing potential changes in preventive behaviors to be captured over time. We have clarified this point in the revised manuscript.

**Comment 3:** From the description it seems that the 'first documented infection' (n=11,129) was actually the number of participants with a single documented COVID-19 infection. Similarly, the 'second infection' (n=890) were patients with two documented infections & 'third-time infection' (n=32) were patients with three documented infections. If so, kindly mention it explicitly to avoid ambiguity.

**Response:** We appreciate the reviewer’s observation. The analysis included 12,051 cases of COVID-19. Among these, 11,129 (92.3%) corresponded to individuals’ first documented infection, 890 (7.4%) to second documented infection (reinfections), and 32 (0.3%) to third documented infection. Therefore, a single participant could contribute to more than one category (first, second, or third documented infection). In addition, because epidemiological and clinical data were collected at each testing event, the same participant could provide updated information on risk factors and comorbidities over time. We have revised the manuscript to make this point explicit.

**Comment 4:** It will be interesting to examine if certain viral variants were more prone to reinfection than others. The authors seem to have the requisite data for this analysis.
**Response:** We thank the editor for this suggestion. In response, we have expanded the Results section to present reinfection rates stratified by viral variant, both as proportions within each epidemic wave and as incidence per 10,000 inhabitants. Reinfections were estimated at 4.55 (95% CI: 2.56–6.55) during the Gamma wave, 9.34 (95% CI: 6.48–12.20) during Delta, 67.41 (95% CI: 59.73–75.09) during Omicron, and 2.28 (95% CI: 0.87–3.69) during Omicron BA.2 sub-lineage. These findings demonstrate that Omicron was associated with a markedly higher reinfection burden compared to Gamma, Delta, and Omicron BA.2. We also revised the Discussion section accordingly, which is consistent with reports of greater immune escape associated with Omicron lineages.

**Reviewer's Responses to Questions**

**Question 3:** Have the authors made all data underlying the findings in their manuscript fully available (please refer to the Data Availability Statement at the start of the manuscript PDF file)?

The [PLOS Data policy](https://track.editorialmanager.com/CL0/https:%2F%2Fjournals.plos.org%2Fglobalpublichealth%2Fs%2Fdata-availability/1/010f01989d193203-1b93047b-2b3f-4744-bfa3-2925920cb1ff-000000/uchVUGyKHHhiph0MD6DMCenCo_bw5_WIzbQC0dcW_Ic=223) requires authors to make all data underlying the findings described in their manuscript fully available without restriction, with rare exception. The data should be provided as part of the manuscript or its supporting information, or deposited to a public repository. For example, in addition to summary statistics, the data points behind means, medians and variance measures should be available. If there are restrictions on publicly sharing data—e.g. participant privacy or use of data from a third party—those must be specified.

**Response:** We confirm that all data underlying the findings of this study have been made fully available in accordance with the PLOS Data Availability Policy. The dataset has been deposited in the public repository Zenodo and can be accessed at <https://doi.org/10.5281/zenodo.14541548>.

**Reviewer #1:**

**Abstract**: Make sure there are no errors with presentation of results. Ideal scenario use 95% Confidence interval instead of p-values. (Line 40: 95% p<0.001, and Line 44).
**Response:** We thank the reviewer for this observation. We have revised the Abstract to remove the p-values and present the results using 95% confidence intervals only.

**Results**: Slight improvements needed in presentation of results in tables. And additional description for preference of Poisson regression model.

**Response:** We thank the reviewer for this comment. We have revised the presentation of the tables to improve clarity and readability. We selected Poisson regression with robust variance to obtain risk ratios (RR) directly. Reinfection was not extremely rare overall (~8%) and was more frequent in specific waves, so odds ratios could overestimate effects. The robust Poisson approach is appropriate for cohort designs and avoids convergence issues common with log-binomial models. We have expanded the description of the statistical analysis in the Methods section.

**Reviewer #2:**

**Comment 1:** The article is relevant and provides real-world information that allows for considerations in health surveillance for decision-making regarding population protection, and therefore has a global public health aspect. However, part of the information provided and final considerations could be presented in the introduction to clarify to the reader that it is a specific aspect in a municipality with an implemented health surveillance system, but which has particular characteristics.

It is suggested to change the title, given that clinical and epidemiological aspects were not deeply explored, but rather demographic and socioeconomic aspects as characteristics associated with SARS-CoV-2 re-infection.

**Response:** We thank the reviewer for this comment. We have revised the title as suggested and expanded the Introduction section to emphasize that the study was conducted in Serrana, State of São Paulo, Brazil, a mid-sized municipality with a strengthened epidemiological surveillance system and specific socioeconomic characteristics, which provides a specific context for the findings.

**Comment 2:** Characterisation of the municipality of Serrana in the interior of the State of São Paulo, Brazil: Given that demographic characteristics were mentioned, a greater characterisation of the municipality of Serrana is warranted, being a small town in the interior of a state where rural activities seem to be relevant, as well as its proximity to a larger hub municipality. Thus, a more adequate characterisation would also involve the flows related to the relationships between these municipalities.

**Response:** We thank the reviewer for this helpful suggestion. Serrana is a town with a population of 43,909 inhabitants in 2022, according to an official and compulsory national census. The municipality has a mixed economy, with relevant agricultural activities (mainly sugarcane cultivation), agroindustrial production, commerce, and services. Serrana is considered a commuter town, as a large number of residents work or study in the neighboring city of Ribeirão Preto, a major urban and economic hub located approximately 20 km away. This daily mobility between Serrana and Ribeirão Preto reflects close socioeconomic ties and may have influenced both exposure to SARS-CoV-2 and access to healthcare during the pandemic. We have expanded the description of Serrana in the Methods section.

**Comment 3:** Improve Keywords: The keywords do not facilitate the search for the real theme of the article, such as demography, pandemic, health surveillance, and vaccination.
**Response:** We thank the reviewer for this observation. The keywords have been revised to better reflect the scope of the study.

**Comment 4:** Line 70: The reference presented in line 70 regarding surveillance in the municipality of Serrana, written by the same primary author, does not show the DOI - <https://doi.org/10.1016/j.puhip.2022.100301>, which would facilitate finding the article that, as verified, is closely related to the submitted manuscript.

**Response:** We thank the reviewer for this observation. The reference has been updated to include the DOI to facilitate access.

**Comment 5:** In line 147, the wording should be reconsidered, given that both continuous and discrete variables have a quantitative aspect.

**Response:** We thank the reviewer for this observation. The sentence was revised to clarify that both continuous and discrete variables were considered quantitative, while categorical variables were described separately.

**Comment 6:** Lines 149 and 150: Clarification is suggested regarding the term qualitative, given that, although it is a qualifier, the reader may have doubts whether the proposal will bring qualitative information related to this type of research, which is not the case. It is a study with a quantitative and descriptive aspect.

**Response:** We thank the reviewer for this clarification. To avoid confusion, the wording was revised: the term “qualitative variables” was replaced with “categorical variables,” since the study is quantitative and descriptive in nature.

**Comment 7:** Furthermore, the term gender was repeatedly used in the text; it would be appropriate to consider using the term sex, given that issues related to gender are not addressed, but rather the characterisation between male and female.

**Response:** We thank the reviewer for this observation. The term “gender” has been replaced with “sex” throughout the manuscript to more accurately reflect the variable used.

**Comment 8:** Given the different statistical methods used, it is suggested to create a table that presents method versus purpose of use.

**Response:** We thank you for this comment. We have clarified the statistical methods used and have opted to retain a more detailed description of them and their respective purposes in the Methods section.

**Comment 9:** Another important aspect is to make it clear that the waves and months referred to relate to the identification of variants according to the surveillance system implemented in the municipality used as a reference, and not what was identified in the international scenario.

**Response:** We thank the reviewer for this comment. We have revised the Methods section to clarify that the definition of variant “waves” was based on genomic sequencing data from the local surveillance system, and that these periods reflect the local context in Serrana rather than international timelines.

**Comment 10:** Given the same ethics committee approval code between the presented manuscript and the work pointed out by the main author in the bibliography, clarification is needed regarding the ethics committee's affiliated institution. Clearly state the committee's affiliation.

**Response:** We thank the reviewer for this observation. We have clarified in the revised manuscript that the affiliated institution of the ethics committee is the Medical School of Ribeirão Preto, University of São Paulo (FMRP/USP). The same ethics approval code was used in a previously published manuscript because both studies are derived from the same surveillance database, although they addressed different objectives (implementation of the system versus reinfection analysis).

**Comment 11:** In line 207, the situation where the use of the term sex and not gender is suggested is well illustrated, given that it refers to the differentiation between male and female.
**Response:** We thank the reviewer for this observation. The term “gender” has been replaced with “sex”, as suggested.

**Comment 12:** Line: 145: In terms of wording and for the terminology to be well framed in genomic characterisations, it should be noted that BA.2 refers to a sub-lineage of the Omicron variant.

**Response:** We thank the reviewer for this observation. The text has been revised to clarify that BA.2 refers to a sub-lineage of the Omicron variant.

**Comment 13:** Insert DOI in the main author's reference - Ferreira NN, Garibaldi 358 PMM, Moraes GR, Moura JC, Klein TM, Machado LE, et al.
359 The impact of an enhanced health surveillance system for COVID-19 management
360 in Serrana, Brazil. Public Health in Practice. 2022;4.

**Response:** We thank the reviewer for this observation. The reference has been updated to include the DOI, as suggested.

We thank the editor and reviewers for their constructive comments, which helped us improve the clarity and quality of the manuscript.
