# Supplementary material for: Demographic and socioeconomic characteristics associated with SARS-CoV-2 reinfection: An observational study
Source: PLOS Glob Public Health. 2026 Mar 10;6(3):e0006103. doi: 10.1371/journal.pgph.0006103 (PMC12974802; doi:10.1371/journal.pgph.0006103)
Supplement: Attachment — Submitted filename: Response_to_Reviewers_auresp_2.docx [file pgph.0006103.s008.docx]

December 21, 2025

Dr. Rajiv Sarkar

Academic Editor

*PLOS Global Public Health*

Re: PGPH-D-24-03019 - Clinical, epidemiological, and socioeconomic characteristics associated with SARS-CoV-2 reinfection: an observational study.

Dear Dr. Sarkar,

We thank you and the referees for the review of our manuscript. We have made the necessary changes to a new version of the manuscript accordingly. The answers and changes are detailed below and are highlighted in the manuscript.

**Editor Comments:
Requirement 1:** From the description in lines 219-222, there were 922 documented reinfections (890 second infections and 32 third infections), which would result in a reinfection rate of 7.6% (922/12051) and not 8.28%, as mentioned. Kindly cross-check.

**Response:** Thank you for this observation. In the original version, we calculated the reinfection rate using the number of individuals at risk after excluding those who eventually experienced reinfection (n = 11,129), which resulted in a higher estimated reinfection rate (8.28%). We agree that the reinfection rate should instead be calculated using the total number of SARS-CoV-2 cases observed during the study period (n = 12,051) as the denominator. Accordingly, we have corrected the reinfection rate to 7.6% (922/12,051), in line with the standard definition and your suggestion. We appreciate your careful review and have updated the manuscript accordingly.

**Requirement 2:** For the details on the severity of SARS-CoV-2 reinfections and viral variants (lines 270-294), kindly specify how many observations were available for the analysis as, from Table 3, it seems that the data was available for a very small subset of cases (330 with single infection and 15 with two or more infections, i.e., a total of 345). Also, the numbers in Table 3 do not match with that presented in lines 283-286 on the number of reinfections by viral variant, which adds up to 374 reinfections (20 + 43 + 301 + 10). Why this discrepancy?

**Response**: Thank you for this observation. We understand the source of the confusion and appreciate the opportunity to clarify this point. The apparent discrepancy arises because the text in lines 270–294 refers to two different populations.

The first paragraph, which precedes Table 3, describes only the subset of severe cases (330 primary infections and 15 reinfections; total = 345). The second paragraph reports the distribution of reinfection episodes according to the predominant viral variant in each epidemiological wave (20 + 43 + 301 + 10 = 374). The remaining 548 reinfections occurred during epidemiological weeks in which there was no clear predominance of a single viral variant. This second analysis is independent of disease severity and therefore does not correspond to the data presented in Table 3.

To avoid further confusion, we added a new subtitle to this section and revised the opening sentence of the second paragraph to explicitly state that it refers to a separate analysis based on the entire reinfection dataset. We appreciate your careful reading and hope this clarification resolves the issue.

**Requirement 3:** How was the incidence of reinfections by viral variants (lines 289-292) calculated? From the data presented in lines 283-286, the highest percentage of reinfections (13.7%) were observed in cases with the Omicron BA.2 sub-lineage, although the incidence of reinfection in that variant was lower than the Delta or the Omicron variant, both of which had lower percentage of reinfections (2.3% and 7.9% respectively). Kindly cross-check.

**Response:** Thank you for this comment. The percentages reported in lines 283–286 (e.g., 1.0%, 2.3%, 7.9%, and 13.7%) represent the proportion of reinfection episodes among all SARS-CoV-2 cases within each viral variant wave (for example, 10 reinfections among 73 SARS-CoV-2 cases during the Omicron BA.2 sub-lineage wave, corresponding to 13.7%).

By contrast, the numbers reported in lines 289–292 correspond to population-based incidence rates of reinfection, expressed per 10,000 inhabitants. These incidence rates were calculated by dividing the number of reinfection episodes observed during each variant wave by the resident population of the study area. Therefore, it is expected that Omicron BA.2 shows the highest proportion of reinfections among SARS-CoV-2 cases in that wave (because the total number of cases was small), while still presenting a lower population-based incidence compared with the Delta and Omicron waves, which involved a much larger absolute number of cases and reinfection episodes.

We have revised the manuscript to clarify the denominators used in each analysis and to explain this difference in interpretation.

**Requirement 4:** Please round the percentages to one decimal place. The RRs and 95% CIs can be rounded to two decimal places, and p-values to three decimal places.

**Response:** We thank the editor for the guidance. All percentages have now been rounded to one decimal place, relative risks and 95% confidence intervals to two decimal places, and p-values to three decimal places, as requested. The tables and text have been updated accordingly.

**Requirement 5:** Please thoroughly proof-read the manuscript as there are a few typos.
**Response:** We appreciate the editor’s observation. The manuscript has been thoroughly proof-read, and all typographical errors identified have been corrected.

**Reviewers' comments:**
**Reviewer #1**

**Comment 1.** Authors mentioned that females were at greater risk than males for reinfection. In the discussion there are reasons given as to why that is the case. Sex is not a modifiable risk factor, however activities potentially done by women and not men may be the reason for reinfections. These could be the areas of public health interventions.

**Response:** Thank you for this insightful comment. We agree that sex itself is not a modifiable risk factor and that the higher reinfection rate observed among females is likely related to differential exposure patterns and testing behavior rather than biological differences. In response, we have revised the Discussion to more directly address potential mechanisms underlying this association, such as higher test uptake, caregiving roles, and occupational or social exposure, which may represent meaningful targets for public health interventions.

**Comment 2.** Line 43-46 prevalence was presented as a relative risk. Could this be a typo?

**Response:** Thank you for pointing this out. Yes, this was indeed a wording error. We were not referring to prevalence, but to the risk of progressing to moderate or severe disease, which was appropriately expressed as a relative risk. The sentence has been corrected to reflect this

**Comment 3.** Line 186-188, since this was a cohort study during COVID, Odds ratios would not have been appropriate based on the study design as well as the fact that COVID-19 infection and reinfection were not rare outcomes during the period of investigation. Perhaps it is not necessary to justify RR over OR.

**Response**: Thank you for this comment. We agree that, given the cohort design and the high frequency of COVID-19 infection and reinfection during the study period, the use of relative risks is appropriate and does not require extensive justification. This explanatory sentence was added in response to a previous reviewer who specifically requested clarification regarding our choice of RR over OR. We have removed this justification.

**Comment 4.** If you can improve the discussion by being more direct about why risk factors were seen as such. At the moment it looks like a comparison of your results and those of other studies.

**Response:** Thank you for this constructive suggestion. We agree that the Discussion section could more clearly articulate the potential mechanisms underlying the associations identified in our regression analyses. Our intention was indeed to evaluate demographic and socioeconomic factors as potential risk factors for reinfection through Poisson regression with robust variance, first in crude analyses and then in adjusted models. In response to your comment, we have revised the Discussion to provide a more direct interpretation of why specific variables may act as risk factors, beyond simply comparing our findings with those of previous studies. This includes a clearer explanation of possible behavioral, exposure-related, and structural factors that could help explain the observed associations.

We thank the editor and reviewers for their constructive comments, which helped us improve the clarity and quality of the manuscript.
