# Supplementary material for: Demographic and socioeconomic characteristics associated with SARS-CoV-2 reinfection: An observational study
Source: PLOS Glob Public Health. 2026 Mar 10;6(3):e0006103. doi: 10.1371/journal.pgph.0006103 (PMC12974802; doi:10.1371/journal.pgph.0006103)
Supplement: Attachment — Submitted filename: Response_to_Reviewers_auresp_3.docx [file pgph.0006103.s009.docx]

February 9, 2026

Dr. Rajiv Sarkar

Academic Editor

*PLOS Global Public Health*

Re: PGPH-D-24-03019 - Clinical, epidemiological, and socioeconomic characteristics associated with SARS-CoV-2 reinfection: an observational study.

Dear Dr. Sarkar,

We thank you and the referee for the review of our manuscript. We have made the necessary changes to a new version of the manuscript accordingly. The answers and changes are detailed below and are highlighted in the manuscript.

**Editor Comments:**

1. Tables 2 and S2: Please add a footnote describing how the adjusted RRs were calculated. Additionally, did you consider conducting a multivariable analysis to identify the key risk factors for reinfection, while adjusting for potential confounders?

**Response**: Thank you for this comment. We have added a footnote to Tables 2 and S2 clarifying that adjusted relative risks were estimated using multivariable Poisson regression with robust variance. We performed a multivariable Poisson regression with robust variance, adjusted for potential confounders, including demographic, socioeconomic, clinical, and behavioral covariates. Accordingly, we revised the Statistical Analysis section of the manuscript to clarify that a multivariable analysis was performed and to describe the covariates included in the multivariable models.

2. Table 3: Please include a footnote specifying the statistical tests used to calculate the p-values. Given the small number of cases with multiple (two or three) infections, were the assumptions underlying the statistical tests met?

**Response**: Thank you for this comment. We have added a footnote to Table 3 specifying the statistical tests used to calculate the p-values. Age was compared using the Student’s t-test, while categorical variables were analyzed using Fisher’s exact test.

Given the small number of severe cases with multiple infections, Fisher’s exact test was specifically chosen for categorical variables, as it does not rely on large-sample approximations and is appropriate when expected cell counts are low. For age, the distribution was approximately symmetric, and the t-test was considered suitable; however, we acknowledge that the limited number of cases with multiple infections reduces statistical power.

We have clarified in the manuscript that these analyses are exploratory and that conclusions regarding severity among individuals with multiple reinfections should be interpreted with caution due to the small sample size.

3. Table S1: Please replace the term “Gender” with “Sex,” to conform with the terminology used in Tables 1–3. Additionally, categorize participants as “Females” and “Males” rather than “Feminine” and “Masculine”.

**Response**: We thank the editor for the guidance. The table have been updated accordingly.

4. Severity of SARS-CoV-2 reinfections: Did the severity of reinfections differ by the viral variant, particularly considering the fact that the Delta variant was more pathogenic than Alpha or Omicron variants?

**Response**: Thank you for this important question. We agree that differences in pathogenicity across variants are highly relevant from a clinical perspective. The primary objective of the present study was to evaluate the risk of SARS-CoV-2 reinfection and its associated factors at a population level, rather than to assess clinical severity or disease outcomes across different viral variant waves. Consequently, the study was not powered to support formal comparisons of severity outcomes by variant.

To address this point, we have now added descriptive information on severe reinfection cases across variant waves to the Results section.

**Reviewers' comments:**

1. Line 250-252: Is this the correct way of interpreting. if 6-9 minimum wages is the baseline, then compared to 3 minimum wages the risk is lower? can you double check on this and other interpretations for the same variable.

**Response**: Thank you for raising this point. After carefully reviewing the manuscript, we confirmed that the text in lines 250–252 is purely descriptive and refers only to the prevalence of income categories in the study population, without any interpretation of risk. However, we identified that the potential source of confusion was a wording issue in line 273-275, where the direction of the association between income categories and reinfection risk was not clearly stated. We have revised this sentence to accurately reflect the adjusted analysis, clarifying that individuals with a family income of up to three minimum wages had a lower risk of reinfection compared to those earning six to nine minimum wages (RR 0.53, 95% CI 0.36–0.77; p = 0.001). No changes to the statistical analyses were necessary.

2. In the excel file, it looks like some under variable number of people on household it looks like some people's names are in that column. I uploaded the csv file into STATA and tabulated the variable 'peoplelivinginthesamehouse'. Please double check on this and potentially reanalyze your data.

**Response**: Thank you for carefully checking the dataset and for pointing this out. We reviewed the original Excel file and confirmed that, in a small number of rows, the variable “people living in the same household” contained text entries (names) instead of numeric values. These entries have now been corrected, and the updated dataset has been uploaded to the platform Zenodo (10.5281/zenodo.18474588).

Importantly, the statistical analyses were conducted using complete-case analysis, including only participants with valid data for all variables included in each model. Therefore, records with non-numeric or missing values for this variable were automatically excluded from the analyses and did not contribute to the statistical results. Consequently, correcting these entries does not change the analytical sample nor the results previously reported, and reanalysis was not required.

3. Line 84-85: Were the coordinates necessary? Unless you wanted to do a GIS type of analysis?

**Response**: Thank you for the comment. We agree that the geographic coordinates are not necessary, as no GIS-based analyses were performed. The coordinates have therefore been removed from the manuscript.
