# Supplementary material for: Demographic and socioeconomic characteristics associated with SARS-CoV-2 reinfection: An observational study
Source: PLOS Glob Public Health. 2026 Mar 10;6(3):e0006103. doi: 10.1371/journal.pgph.0006103 (PMC12974802; doi:10.1371/journal.pgph.0006103)
Supplement: Attachment — Submitted filename: Response_to_Reviewers_auresp_4.docx [file pgph.0006103.s010.docx]

February 21, 2026

Dr. Rajiv Sarkar

Academic Editor

*PLOS Global Public Health*

Re: PGPH-D-24-03019 - Clinical, epidemiological, and socioeconomic characteristics associated with SARS-CoV-2 reinfection: an observational study.

Dear Dr. Sarkar,

We sincerely thank you and the reviewer for the careful evaluation of our manuscript. We have revised the manuscript accordingly and addressed each point in detail below. All modifications have been clearly indicated in the revised version of the manuscript.

Additionally, we have updated reference number 8. In the previous version, this reference corresponded to a preprint; however, the study has since been formally published, and we have revised the citation to reflect its final published version.

**Editor Comments:**

1. Tables 1 and 2: Monthly family income – please mention the $-value for each category in parenthesis or as footnote, similar to what has been presented in S1 Table.

**Response**: Thank you for this suggestion. We have revised Tables 1 and 2 to include the corresponding dollar values for each monthly family income category.

2. Discussion, lines 365-366: ‘Then, the vaccination scheme might have influenced the clinical outcomes of patients with COVID-19 reinfections.’ Should lines 382-385 not immediately follow this sentence, with an explanation of how the COVID-19 reinfections may have been influenced by the vaccination scheme and previous infections?

**Response**: Thank you for this insightful suggestion. We agree that a clearer integration of these concepts would improve the flow of the Discussion. Accordingly, we have reorganized the paragraph so that the explanation regarding how previous infections and vaccination may have influenced the clinical outcomes of reinfections now immediately follows the statement referring to the vaccination scheme.

3. Figure 2: Please consider adding a second y-axis for 2nd and 3rd infections as their numbers are too small compared to the number of 1stinfections.

**Response**: Thank you for this valuable suggestion. We have revised Figure 2 to include a secondary y-axis for second and third infections, as their absolute numbers were substantially lower than those of first infections. This modification improves the visualization and allows a clearer comparison of trends across infection episodes.
